# Epidemiology of Multidrug-Resistant *Salmonella* Typhi and Paratyphi Isolated From Stool Culture

**DOI:** 10.1155/jotm/3480080

**Published:** 2024-12-05

**Authors:** Tito Aloys Ndima Etouke, Georges Ful Kuh, Boris Emmanuel Djoumsie Gomseu, Vanessa Linda Nzesseu, Jean-De-Dieu Tamokou, Jean Paul Dzoyem

**Affiliations:** Department of Biochemistry, University of Dschang, Dschang, Cameroon

**Keywords:** enteric fever, multidrug resistance, risk factors, *S.* Paratyphi, *S.* Typhi

## Abstract

Enteric fever is a significant health problem in developing countries caused by *Salmonella enterica* serovars Typhi and Paratyphi. Unfortunately, the burden of the disease remains high not only because of the complications related to the disease but also, especially, because of the spread of the strains of *Salmonella* resistant to antibiotics. The aim of the present study was to evaluate the antibiotic resistance patterns of *Salmonella* Typhi and Paratyphi clinical isolates as well as the risk factors associated with infection. This cross-sectional study was conducted from June 2020 to September 2021. One thousand and seventy-six patients in the age range (1− ≥ 50 years) were recruited including 423 (39.31%) infected with *S.* Typhi, 115 (10.68%) infected with *S.* Paratyphi, and 538 (50%) noninfected after obtaining their informed consent using a face-to-face interview and questionnaire. The stool samples were collected in clean and sterile boxes reserved for this purpose and were cultured. Demographic parameters such as sex, age, occupation, water source, level of education, as well as clinical signs and symptoms were obtained. The resistance profile determination was carried out by the disk diffusion method. A multivariate logistic regression analysis was performed to identify factors associated with infection. Results of multivariate logistic regression analysis showed positive and significant associations (OR > 1; *p* < 0.05) between enteric fever and women among the age groups: 1–10 years, 11–20 years, and 21–30 years. These positive associations were also noted in patients who ate shellfish, salads, fruits, and vegetables; in patients who consumed ice cubes; as well as those who consumed food and drinks offered by ambulant merchants. This indicated that they are more likely to be infected by *S. enterica* than others. The level of multidrug-resistant (MDR) *S. enterica* to first-line antimicrobial agents ampicillin, chloramphenicol, and co-trimoxazole was high and selectively distributed according to age groups, marital status, profession, level of education, source of water, and lifestyle. The results highlighted the emergence of MDR *S. enterica* isolated in the study population, demonstrating resistance to first-line drugs, fluoroquinolones, and third-generation cephalosporins. Further studies with large-scale samples are needed to validate the present results and to monitor MDR *S.* Typhi and *S.* Paratyphi serovars in other parts of Cameroon.

## 1. Introduction

*Salmonella enterica* infection remains a public health and economic problem in industrialized and underdeveloped countries with the high costs associated with disease surveillance, prevention, and treatment [1]. *Salmonella enterica* is a major cause of invasive infections worldwide [2, 3]. Enteric fever (EF) also called typhoid fever (TF) and paratyphoid fever (PTF) or simply typhoid is a bacterial disease transmitted by the fecal–oral route [4, 5], via contaminated water or food. In addition, TF is estimated to cause approximately 11–20 million cases and 1,28,000–1,61,000 deaths, while PTF causes 6 million cases and 54,000 deaths annually worldwide [6]. Despite the decrease in the morbidity and mortality rate associated with this infection in industrialized countries, TF remains as one of the main public health problems in developing countries [5, 7]. In 2002, TF was the cause of hospitalization of 4,08,837 cases in Africa [8]. However, despite control measures deployed against the infection, the impact of the disease is made more complicated due to an ever-increasing urgency of antimicrobial resistance, including multidrug resistance (MDR) in *S.* Typhi for commonly prescribed antibiotics [9, 10]. MDR in *Salmonella*, defined as combined resistance to ampicillin, chloramphenicol, and co-trimoxazole, emerged in the 1970s, and resistance to newer antibiotics including fluoroquinolones, third-generation cephalosporins, and azithromycin has been accumulating over the last few decades [1]. Moreover, antimicrobial resistance is widespread and patients treated with ineffective antimicrobials show poor clinical response and a high rate of complications and death [11]. Internationally, there is growing concern about antimicrobial resistance, which is currently estimated to cause more than 700,000 deaths per year worldwide. If no appropriate action is taken to stop its progress, antimicrobial resistance will cost an estimated 10 million lives by 2050 [12]. In 2016, a large outbreak of extensively drug-resistant (XDR) TF linked to contaminated water began in Sindh Province, Pakistan, and continues today [13, 14]. XDR Typhi infections among travelers to or from Pakistan have been reported globally, including in the United States [15]. Indeed, the production of carbapenemase constitutes the most powerful mechanism of resistance of enterobacteria to carbapenems [16]. The combination of improvements in water, sanitation, and hygiene (WASH) and vaccination using safe and efficacious vaccines will be pivotal for TF prevention and potential elimination in endemic countries [17]. As such, immunization with typhoid conjugate vaccines (TCVs), combined with building upon achievable and already existing improvements in household WASH, may be an effective strategy for typhoid prevention and potential elimination in hyperendemic urban slums [18].

According to some studies, the resistance of *S. enterica* to antimicrobials is a barrier to effective treatment of a growing range of infections and requires action, especially as we face the emergence of new resistance mechanisms that are rapidly spreading around the world [19]. In addition, a previous study stressed the presence and the spread of MDR salmonellosis in the Bamenda District Health area, Cameroon, where there was a noticeable re-emergence of chloramphenicol-susceptible *Salmonella* [20]. Prescription and utilization of antibiotics in Cameroon in general and in Dschang Health District in particular are suboptimal with reported antibiotic resistance frequently identified in quinolones [21]. Many parts of Cameroon still lack the facilities for stool culture and antibiotic susceptibility testing which clearly leads to missing MDR isolates. Delayed reporting of MDR *Salmonella enterica* is associated with prolonged hospital stay, high healthcare costs, and increased morbidity and mortality [22]. This demonstrates a consistent and continuous menace of resistance to antibiotic therapy. Information on prevalent levels of antibiotic resistance among *Salmonella enterica* is useful in making an appropriate choice of antibiotic treatment without aggravating the illness and preventing antibiotic resistance.

It is therefore necessary to continuously update the recommendations on the treatment because of the development of resistance to the therapeutic substances generally used. A study is therefore necessary to establish the current resistance profile of *S. enterica* to antimicrobials, which could be used in the better control and management of this infection. The aim of this study was to evaluate the resistance profile of *Salmonella enterica* isolates in patients at the Dschang Annex Regional Hospital (DARH).

## 2. Materials and Methods

### 2.1. Design, Study Population, and Sample Size Determination

This was a cross-sectional study conducted at DARH, West Region, Cameroon during the period from June 2020 to September 2021. The DARH is one of the well-equipped hospitals in the Western Region of Cameroon. The participants of this study were those suspected of EF with symptoms such as fever, headache, fatigue, and anorexia and those with no EF. Male and female participants with no age restriction were selected for the study. Inclusion criteria were participants with the complaints of fever and other gastrointestinal tract symptoms (nausea, vomiting, abdominal pain, diarrhea, and constipation) of TF viz, who signed the informed consent form and the assent for those under 21 years. For the noninclusion criteria, we withdrew participants who refused to sign the informed consent and patients on antibiotic treatment whose action spectrum included the bacteria *Salmonella enterica* serovars Typhi and Paratyphi. Finally, we dropped out participants who had finished a course of antibiotic therapy within the last 2 weeks' study because this would reduce the viability of the bacteria which can no longer multiply effectively and therefore its level would be reduced in the sample thus making its isolation difficult.

The prevalence of 30.10% from a prior study [16] was used to calculate the sample size using the Thrusfield [23] formula at a 5% level of significance and a 95% confidence interval.

(1) $$\begin{array}{c} n=\frac{t^{2}\times p\left(1-p\right)}{m^{2}}, \end{array}$$

where *n* is the required sample size, *t* is the confidence level at 95% (standard value of 1.96), *p* is the expected prevalence, and *m* is the desired absolute precision.

Based on the above formula, the minimum sample size was 324 participants. However, 1076 participants were recruited. Age-matching for noninfected participants was not considered.

Stool samples were collected from 1076 participants who agreed to give their consent to participate in the study. Stool culture was used to stratify participants into infected cases and noninfected ones and subdivided into 3 groups: 423 *Salmonella* Typhi positive patients, 115 *Salmonella* Paratyphi positive patients, and 538 *Salmonella* negative participants.

### 2.2. Stool Collection

The stool sample (25 g) was collected in well-labeled sterile plastic containers from each participant for the identification of *S. enterica*–positive patients, and treated using well-defined standard protocols [24]. Sociodemographic and clinical examination data were collected through a face-to-face interview with a structured questionnaire.

### 2.3. Bacterial Isolation and Identification

Specimens (3-4 g) were poured in buffered peptone water (BPW, 10 mL) at 37°C for a 24 h pre-enrichment step, followed by a 24 h selective enrichment in Rappaport–Vassiliadis (RV) broth at 41.5°C before streaking on selective media: *Salmonella Shigella* agar (SSA) and Hektoen agar. Then, the cultural and morphological characteristics of the isolates were studied and the isolates were duly identified following three confirmatory biochemical tests, the triple sugar–iron agar (TSI), Simmons citrate test, and the urease [25]. Then, for the study of other biochemical characteristics, the oxidase test, the catalase test, and the API 20E gallery (Bio-Mérieux®) were used [26]. The presumptive *Salmonella* colonies were directly stabled into the TSI agar slant. The inoculated samples were incubated with the caps loosened for 24 h at 35°C. For the urease test, 2 loopful of pure and well-isolated *Salmonella* colonies were inoculated into the urea broth. The inoculated tubes were gently shaken and incubated with the caps loosened for 48 h at 35°C in an incubator. The TSI agar was checked for hydrogen sulfide (H_2_S) production and alkalinity, while the urease test was checked for urea degradation in urea broth [27]. The *Salmonella* colonies that were positive for H_2_S gas on TSI agar and urease negative were subcultured onto the fresh *SS* agar plates. Finally, a single colony was transferred into 20 mL of nutrient broth (enrichment medium) and incubated for 18 h at 35°C for further studies. The serotyping of the *Salmonella enterica* isolates was performed according to Kauffman–White scheme [27]. Furthermore, polyvalent *Salmonella* antisera Phase 1 and Phase 2 flagellar H antigens were used for serovars determination of the *Salmonella* isolates [28].

### 2.4. Determination of Resistance Profile

Resistance profiling of *Salmonella enterica* isolates was performed using the modified Kirby–Bauer disk diffusion method according to the guidelines set forth by the Clinical and Laboratory Standard Institute (CLSI) [29]. The antibiotics were selected according to CLSI guidelines [30]. The antibiotic discs were amoxicillin (20 μg), amoxicillin + clavulanic acid (20 μg), ampicillin (10 μg), ceftriaxone (30 μg), cefotaxime (30 μg), ciprofloxacin (5 μg), levofloxacin (5 μg), perfloxacin (5 μg), ofloxacin (5 μg), chloramphenicol (30 μg), gentamicin (15 μg), amikacin (30 μg), streptomycin (10 μg), tetracycline (30 μg), doxycycline (30 μg), minocycline (30 μg), sulfamethoxazole (23.75 μg), nalidixic acid (30 μg), trimethoprim (5 μg), and co-trimoxazole (1.25/23.75 μg). In this method, the broth culture of the organism tested (comparable to McFarland #0.5 tube; inoculum density: 1.5 × 10^8^/mL) was evenly coated on the surface of the Muller–Hinton agar (MHA). Then, the antibiotic discs were placed on inoculated MHA and incubated at 37°C for 18–24 h. After incubation, the diameters of the zones of inhibition were measured and the results were interpreted as “resistant,” “intermediate,” or “susceptible” to this antibiotic on the basis of the CLSI guidelines [31, 32]. *Salmonella enterica* isolates with resistance to three or more classes of antimicrobial agents were considered to be MDR *S. enterica* [33].

### 2.5. Ethical Consideration

The protocol of this study was approved by the National Ethics Committee of the Research for Human Science (CNERSH), Yaoundé, Cameroon (ref: 2020/11/73/CE/CNERSH/SP) coupled with the authorization of the District Hospital of Dschang. Thus, before data collection, participants were clearly informed about the objectives of the study and the experimental protocol. Those who agreed to participate signed the informed consent form, and the consent of all parents of minor participants was obtained. A structured questionnaire was administered to each participant to assess the sociodemographic and economic status [34].

### 2.6. Statistical Analysis

The data generated through laboratory analysis were inserted in Microsoft Excel 2016 software. The data were analyzed using the statistical software for the social sciences (SPSS) Version 22. The chi-square (*χ*^2^) test was performed to compare categorical variables, with statistical significance at *p* < 0.05. Descriptive statistics were used to describe the sociodemographic characteristics and antimicrobial resistance. A multivariate logistic regression analysis (formula used: *Y* = *β*_0_ + *β*_1_ *X*_1_ + *β*_2_ *X*_2_ + …*β*_*n*_*X*_*n*_ + μ) was carried out to determine the factors associated with infection. Predictor variables were sex, age range, marital status, profession, level of education, source of water, lifestyle, consumption of food and drinks offered by ambulant merchants, digestive disorders, stomach pain, constipation, diarrhea, loss of appetite, fever, headache, sore throat, cough, extreme tiredness, bleeding from the rectum, pink spots, diarrhea, and constipation. Outcome variables were *Salmonella* Typhi and *Salmonella* Paratyphi infections, whereas controlled variables were male, participants ≥ 51 years, widowers, civil servants, patients having university level, and those who drank other sources of water. The significance threshold was 5%.

## 3. Results

The frequencies of *Salmonella* Typhi and Paratyphi infections according to the sociodemographic and clinical characteristics of the study population are depicted in Table 1. The mean age of 1076 study volunteers of both sexes was 25.29 ± 12.51 years (mean ± standard deviation). The prevalence of *S.* Typhi infection (39.31%) was higher compared to that of *S.* Paratyphi serovars infection (10.68%). In addition, a high prevalence of *Salmonella* Typhi and Paratyphi infections was observed in women compared to men in the study population. The frequencies of *S.* Typhi and *S.* Paratyphi infections were significantly highest among participants with the age range of 11–20 and 21–30 years, indicating that these age groups are more likely at risk of infection. The highest frequencies of both *S.* Typhi and *S.* Paratyphi infections were observed in single participants followed by those living in cohabitation and those who are married. Patients having university and secondary educational levels were the most infected in the study population compared to illiterates and those having a primary level of study. With regard to profession, the prevalences of *S.* Typhi and *S.* Paratyphi infections were raised among pupils, students, and those working in the informal sector. With respect to the source of water, participants who drank tap water and well water were the most infected compared to those who drank borehole water and other sources. According to lifestyle, the prevalences of *S.* Typhi and *S.* Paratyphi infections were highest in participants who ate crustaceans, salads, fruits, and vegetables; those who consumed food and drinks offered by ambulant merchants; patients who ate with their hands; as well as those who consumed ice cubes. Finally, digestive disorders, fever, abdominal pain, constipation, diarrhea, loss of appetite, headache, cough, and extreme exhaustion were the main signs and symptoms of TF and PTF.

Results of multivariate logistic regression analysis showed positive and significant associations (OR > 1; *p* < 0.05) between *S*. Typhi infection and women; those in the age groups of 1–10 years, 11–20 years, and 21–30 years; patients eating shellfish, salads, fruits, and vegetables; patients consuming ice cubes; and those consuming food and drinks offered by ambulant merchants (Table 2), indicating that they are more likely to be infected by *S.* Typhi than the others. Similarly, positive and significant associations were observed between *S.* Paratyphi infection and women; those in the age group of 1–10 years; patients who ate shellfish, salads, fruits, and vegetables; and those consuming food and drink offered by ambulant merchants (Table 2). However, negative and significant associations (OR < 1; *p* < 0.05) were noted between *S. Typhi i*nfection and married patients, pupils, housewives, patients who drank water from a protected well, patients who washed hands after a bowel movement, and those who ate with hands. Furthermore, married and single patients, housewives, patients who drank water from a protected well and borehole, and patients who ate with hands were less likely (OR < 1; *p* < 0.05) to be infected by *S.* Paratyphi (Table 2). Digestive disorders, stomach pain, constipation, loss of appetite, fever, headache, extreme tiredness, pink spots, diarrhea, and constipation are symptoms significantly more associated (OR > 1; *p* < 0.05) with both *S.* Typhi and Paratyphi infections, whereas cough was significantly more associated (OR > 1; *p* < 0.05) with *S.* Typhi infection (Table 2).

Most *S.* Typhi and Paratyphi isolates were MDR to traditional first-line antimicrobial agents ampicillin, chloramphenicol, and co-trimoxazole (Table 3). A high resistance to newer drugs including nalidixic acid, aminoglycosides, and tetracyclines was also observed (Table 3). The analysis of the results also showed that there was a high susceptibility rate (i.e., > 83%) of *S.* Typhi and *S.* Paratyphi against fluoroquinolones and third-generation cephalosporins. *Salmonella* Typhi isolates were 100% sensitive to pefloxacin, while *S.* Paratyphi showed 100% susceptibility against pefloxacin and ofloxacin (Table 3). A high resistance rate (i.e., above 50%) was observed with *Salmonella* Typhi isolates against amoxicillin, ampicillin (penicillins), gentamycin, amikacin (aminoglycosides), tetracycline, doxycycline, minocycline (tetracyclines), chloramphenicol (phenicols) and sulfamethoxazole (sulfonamides). In addition, *S.* Paratyphi displayed the most resistance rates (i.e., > 50%) against amoxicillin, ampicillin (penicillins), gentamycin, amikacin (aminoglycosides), tetracycline, doxycycline, minocycline (tetracyclines), chloramphenicol (phenicols), sulfamethoxazole, and co-trimoxazole (sulfonamides). *S.* Typhi and *S.* Paratyphi were less resistant to the combination of amoxicillin + clavulanic acid compared to amoxicillin (Table 3). With respect to the quinolone antibiotic class, *S.* Typhi and *S.* Paratyphi were more resistant to nalidixic acid compared to other antibiotics belonging to the same class. Significant differences (*p* = 0.043) were noted between the sensitivity and resistance rates of *S. *Typhi and *S. *Paratyphi against amikacin.

*S*. Typhi isolates were resistant to 3, 4, 5, 6, and 7 antibiotic classes with high resistance rates recorded against 4 and 5 antibiotic classes, whereas *S.* Paratyphi isolates were resistant to 3, 4, 5, and 6 antibiotic classes with a high resistance rate recorded against 4 antibiotic classes (Table 4).

## 4. Discussion

EF remains a public health problem in developing countries due to precarious hygiene conditions and inadequate sanitation [35]. *Salmonella* spp. is one of the most important pathogens that cause food poisoning in Cameroon. Resistance to antibiotics of *Salmonella enterica* serovars Typhi and Paratyphi in developing countries continues to worsen overnight [36] and can lead to treatment failures which would increase the risk of mortality. Although there are no vaccines approved for PTF, WHO recommended the use of the typhoid conjugate vaccine in 2017 for TF [37]. Despite this implementation, we are still witnessing highly resistant/multiresistant *S. enterica* due to the inappropriate use of antibiotics with common self-medication.

In this study, 538 clinical pathogens were isolated and identified as *Salmonella enterica.* We found that the prevalence of typhoid infection (39.31%) was higher than that of paratyphoid (10.68%). These results are in accordance with various global studies where *S.* Typhi was the most dominant serotype among the isolated *S. enterica* strains [38, 39]. In the current study, the prevalence of EF obtained by stool culture was 50%. This result was higher than those obtained nationally in previous studies, especially in Yaoundé (34.5%), Douala (22.5%), Tiko (43%), and Bamenda (8.5%) [38, 39]. This result is also very highly relative to those from studies carried out in India [40, 41] and Nepal [42], which reported prevalences of 0.22%–1.24% and 2.5%, respectively. Likewise, the prevalence of *S.* Typhi infection was higher than that of a study in Egypt with a 5% prevalence [43]. This difference in prevalence could be due to the uneven distribution of *S.* Typhi and *S.* Paratyphi across geographic areas [44] and seasonal variability [45]. In addition, the differences in susceptibility in laboratory detection methods could also explain this difference in prevalence observed. The findings of the present investigation also revealed a higher prevalence of *S.* Typhi compared to that of *S.* Paratyphi. This result is similar to studies carried out in India [40, 46], Indonesia [47], Nepal [48], and Bangladesh [49]. However, few studies in Cameroon and abroad have reported a higher prevalence of *S.* Paratyphi compared to that of *S.* Typhi [50, 51]. Although there is no reason well established for the serotypical variation of EF, the high prevalence of *S.* Typhi could be due to the water transmission of *S.* Typhi because it generally implies smaller inocula than *S.* Paratyphi, with the latter being derived via food transmission which requires greater inocula [52].

Gender analysis showed that the proportion of women with a positive stool culture was much higher than that of men. Hence, the female sex is more vulnerable to being infected than men. This result is in accordance with the study carried out by Ramyil et al. [53] in Jos Metropolis, Nigeria, who found that more women were affected than men. However, this finding is contrary to those observed by Joshi et al. [54] and Gake et al. [55]. It should be noted, however, that a limitation of this analysis is that healthcare-seeking behavior by age and gender was not analyzed. In multivariate logistic regression analysis, our results showed a positive significant association (OR > 1; *p* < 0.05) between age groups, 1–10 years, 11–20 years, and 21–30 years, and EF. This finding is similar to those obtained in the city of Kathmandu [52, 56]. In addition, a previous study conducted in Ngassa, North region of Cameroon, found the prevalence of TF higher in patients under 30 years old and low in patients over 40 years old [57]. Our results partly concur with those obtained in Kinshasa where the age group most affected by the infection was 10–19 years old [58]. Another study conducted in Lebanon showed that the distribution of cases of EF was not homogeneous with the different age groups, and the most affected range was 20–39 years, followed by 10–19 years [59]. The highest noted percentage of the burden of the disease in age groups 11–20 years and 21–30 years can be attributed to their active social life or the habit of eating in restaurants or on the street. The percentage of the burden of the disease observed in children from 1 to 10 years can attributed to environments where inadequate water supply and poor environmental hygiene are problems because of their high level of ignorance.

In addition, the results of multivariate logistic regression analysis showed positive and significant associations (OR > 1; *p* < 0.05) between *S. enterica* infection and patients who ate shellfish, salads, fruits, and vegetables; patients who consumed ice cubes, and those who consumed food and drinks offered by ambulant merchants, indicating that they are more likely to be contaminated than the others. This contamination might have occurred indirectly via water and foods soiled by the stools of asymptomatic patients [60]. This result is consistent with that obtained in a study conducted in Indonesia [47]. However, negative and significant associations (OR < 1; *p* < 0.05) were noted between *S. enterica* infection and married patients, pupils, housewives, patients who drank water from a protected well, patients who washed their hands after a bowel movement, and those who ate with hands, insinuating that the latter are less likely to be contaminated. Furthermore, married and single patients, housewives, patients who drank water from a protected well and borehole, and patients who ate with hands were less likely (OR < 1; *p* < 0.05) to be infected by *S.* Paratyphi. Digestive disorders, stomach pain, constipation, loss of appetite, fever, headache, extreme tiredness, pink spots, diarrhea, and constipation are symptoms significantly more associated (OR > 1; *p* < 0.05) with both *S.* Typhi and Paratyphi infections, whereas cough was significantly more associated (OR > 1; *p* < 0.05) with *S.* Typhi infection. Our observations, to a certain extent, corroborate those of previous studies [58, 61, 62]. Indeed, fever [58, 61, 62], abdominal pain [58, 62], and diarrhea [62] have been found as dominant signs of the TF.

A high sensitivity rate (i.e., > 83%) of *S.* Typhi and *S.* Paratyphi to fluoroquinolones and third-generation cephalosporins was observed. In addition, *Salmonella* Typhi isolates were 100% sensitive to pefloxacin, while *S.* Paratyphi showed 100% sensitivity to pefloxacin and ofloxacin. These results are in agreement with those of Lefebvre et al. [59]. However, the resistance of *S. enterica* against nalidixic acid (28.65%) was remarkable, which is higher than that obtained in Vietnam (19.6%) and lower than those reported in Bangladesh (81.6%) [63] and Kenya (93.2%) [64]. The result of this study also identified lower susceptibility of *S. enterica* against chloramphenicol, which is in agreement with those recorded in Kenya (17.4%) [64], West Africa (38.3%) [65], and Egypt (33%). Also, this finding was supported by the 80.4% resistance in Vietnam [63]. The differences in sensitivity observed with *S. enterica* isolates to available antibiotics may be traceable to certain peculiarities in their genetic constitutions showing that *S. enterica* infections must be properly diagnosed given the fact that two isolates can cause identical symptoms (fever, diarrhea, vomiting, and anorexia) without being affected in the same way by antibiotics.

Antibiotic resistance is now accepted as a major problem in public health and patient care. In the present study, a high rate of antibiotic resistance by *S.* Typhi and Paratyphi isolates was recorded with the commonly used antibiotics. The high percentage of resistance to the commonly used antibiotics could be linked to their prevailing usage and abuse in the area under study and in other unrelated infections [66–68]. In addition, incomplete treatment due to many reasons in developing countries may also be the factor contributing to the development of antibiotic resistance. These results are in agreement with previous reports of a worldwide occurrence of MDR *S. enterica* [69, 70]. A high resistance rate (i.e., above 50%) was observed with *Salmonella* Typhi isolates against amoxicillin, ampicillin (penicillins), gentamycin, amikacin (aminoglycosides), tetracycline, doxycycline, minocycline (tetracyclines), chloramphenicol (phenicols), and sulfamethoxazole (sulfonamides). In addition, *S.* Paratyphi displayed the most resistance rates (i.e., > 50%) against amoxicillin, ampicillin (penicillins), gentamycin, amikacin (aminoglycosides), tetracycline, doxycycline, minocycline (tetracyclines), chloramphenicol (phenicols), sulfamethoxazole, and co-trimoxazole (sulfonamides). These findings to a certain extent substantiate those of previous studies conducted in Nigeria [71], Ethiopia [69], Indonesia [72], Bangladesh [73], and Asia [74, 75]. Indeed, *in vitro* antimicrobial susceptibility testing conducted by Brooks et al. [73] in Bangladesh showed a high resistance level (55.1%) of *S*. Typhi against the first-line drugs, ampicillin, co-trimoxazole, and chloramphenicol (ACC), whereas ceftriaxone resistance was found in isolates from 1 preschool child. Another study conducted in China showed resistance rates of 13.5% and 5.9% of *S.* Typhi and *S.* Paratyphi against ciprofloxacin, 5.4% and 0.8% against levofloxacin, 5.4% and 1.4% against sulfamethoxazole, 10.8% and 2.0% against ampicillin, 5.4% and 0.0% against ceftriaxone, and 0.0% against meropenem and imipenem [76]. A study in Nepal showed a resistance rate of 1.8% of *S.* Typhi against ampicillin and 3.9% of *S. *Paratyphi against ciprofloxacin, while there was no resistance against trimethoprim–sulfamethoxazole [77]. In South Asia, *S.* Typhi and *S.* Paratyphi have been reported to be endemic, and several antibiotics have been used for EF resulting in the development of antimicrobial resistance by these antibiotics [78]. Furthermore, a previous study demonstrated that several *S. enterica* isolates exhibited resistance to the first-line antibiotics used for salmonellosis treatment including ampicillin, trimethoprim–sulfamethoxazole, and chloramphenicol, whereas most isolates were resistant to first- and second-generation cephalosporins and aminoglycosides [74]. Alternative effective antibiotics available for the treatment of these resistant isolates are fluoroquinolones and macrolides [41]. Quinolones are the drugs of choice to treat invasive *Salmonella* infections in the DARH. Carbapenems have also been shown to be active against *Salmonella* species *in vitro* [76]. The decreasing sensitivity observed with quinolones is due to the high resistance rate observed with nalidixic acid. Resistance to nalidixic acid has also been reported in India [79], China [80], and Tunisia [81], whereas previous studies reported decreasing susceptibility to ciprofloxacin among nalidixic acid–resistant *Salmonella* strains due to mutations in the gyrA gene [82] as well as by *qnr* gene acquisition and *parC* mutations [83]. According to our findings, nalidixic acid (quinolones), amoxicillin, ampicillin (penicillins), gentamycin, amikacin (aminoglycosides), tetracycline, doxycycline, minocycline (tetracyclines), chloramphenicol (phenicols), and sulfamethoxazole (sulfonamides) cannot be a potential option for the treatment of EF in the study area due to their resistance. Physicians should be more conscious in prescribing similar antibiotics all over the country.

In this study, it was found that 38.06% of *S.* Typhi and 48.07% of *S.* Paratyphi isolates were MDR. Similar to our results, high MDR *S*. *enterica* clinical isolates were reported in the literature [54, 75, 84] Da Silva et al. [84] in Bangladesh reported 64.28% of MDR *S*. Typhi, whereas reports in Nigeria [51], India [72], and Laos [84] recorded MDR *S*. Typhi rates less than 30%. High MDR *S. enterica* rates to different classes of antibiotics may be due to wrong and inaccurate diagnosis and inappropriate prescription practice of health professionals resulting in the development and spread of MDR strains. This increased multiresistance could also be associated with a rapid transfer of resistance genes among the different species of *Salmonella enterica*. The variation among studies with regard to the proportion of MDR *S.* Typhi and Paratyphi isolates could be linked to the difference in the geographical distribution of isolates, target population, and sample size. This level of multiresistance was taken into account by a meta-analysis study which indicated the worsening trend of antimicrobial resistance in *S.* Typhi and *S.* Paratyphi [36]. It is more troublesome to developing countries since there are limited studies showing a high rate of MDR *Salmonella enterica*. Interestingly, this is a pioneer report to provide evidence for the MDR *Salmonella* Typhi and Paratyphi in Cameroon. In this study, fluoroquinolones and third-generation cephalosporins were the most effective antibiotics and could be the drug of choice for the treatment of infections caused by MDR *S.* Typhi and Paratyphi isolates. Finally, this study proposes a national guideline for antibiotic use against EF in Cameroon. However, this study was not without limitations. Stool culture does not have a better sensitivity but our limited resources could not permit us to do a blood culture which is better in sensitivity [24]. We also regret the lack of healthcare utilization data being incorporated in this study. Moreover, healthcare-seeking behavior across age and gender was not investigated and analyzed. Age-matched noninfected controls were also not included in this study.

## 5. Conclusion

In this study, we report the epidemiology and antibiotic resistance patterns of *S.* Typhi and *S.* Paratyphi isolated in Cameroon. Overall, the results underscore the emergence of MDR *Salmonella enterica* isolated in the study population, demonstrating resistance to first-line drugs, fluoroquinolones, and third-generation cephalosporin. Further studies with large-scale samples are needed to validate the present results and to monitor MDR *S.* Typhi and *S.* Paratyphi in other parts of Cameroon.

## Figures and Tables

**Table 1:** Distribution of study participants according to the *Salmonella* Typhi and Paratyphi status.

Characteristics of participants	Category		Frequency *n* (%) *N* = 1076	*S.* Typhi positive *n* = 423 *n* (%)	S. Paratyphi positive n = 115 n (%)	*Salmonella* negative *n* = 538 *n* (%)	Chi-square (*χ*^2^)	*p* value
Sex	Male		460 (42.75)	167 (39.5)	38 (33.9)	254 (47.2)	9.894	0.007⁣^∗^
	Female		616 (57.25)	256 (60.5)	76 (66.1)	284 (52.8)		

Age range	1–10 years		106 (9.85)	68 (16.1)	8 (7.0)	30 (5.6)	41.031	0.001⁣^∗^
	11–20 years		308 (28.62)	120 (28.4)	38 (33.0)	150 (27.9)		
	21–30 years		379 (35.22)	145 (43.3)	40 (34.8)	194 (36.1)		
	31–40 years		153 (14.21)	56 (13.2)	12 (10.4)	85 (15.8)		
	41–50 years		76 (7.06)	18 (4.3)	10 (8.7)	48 (8.9)		
	≥ 51 years		54 (5.02)	16 (3.8)	7 (6.1)	31 (5.8)		

Marital status	Married		151 (14.03)	40 (9.8)	11 (9.5)	100 (18.6)	26.072	0.001⁣^∗^
	Single		739 (68.68)	309 (73.0)	74 (64.3)	356 (66.2)		
	Concubinage		146 (13.56)	60 (14.2)	23 (20.0)	63 (11.7)		
	Divorced		16 (1.48)	7 (1.7)	2 (1.7)	7 (1.3)		
	Widower		24 (2.23)	7 (1.7)	5 (4.3)	12 (2.2)		

Profession	Students		359 (33.36)	141 (33.3)	37 (32.2)	181 (33.6)	21.888	0.005⁣^∗^
	Pupils		338 (31.41)	155 (36.6)	33 (28.7)	150 (27.9)		
	Housewives		60 (5.57)	23 (5.4)	1 (0.9)	36 (6.7)		
	Informal sector		256 (23.76)	78 (18.4)	37 (32.2)	141 (26.2)		
	Civil servant		63 (5.85)	26 (6.1)	7 (6.1)	30 (5.6)		

Level of education	No schooling		65 (6.04)	31 (7.3)	9 (7.8)	25 (4.6)	17.959	0.006⁣^∗^
	Primary		137 (12.73)	73 (17.3)	10 (8.7)	54 (10.0)		
	Secondary		392 (36.43)	146 (34.5)	43 (37.4)	203 (37.7)		
	University		482 (44.79)	173 (40.9)	53 (46.1)	256 (47.6)		

Source of water	Tap		481 (44.70)	185 (43.7)	54 (47.0)	242 (45.0)	53.350	0.001⁣^∗^
	Protected well		415 (38.56)	131 (31.0)	38 (33.0)	246 (45.7)		
	Drilling (borehole)		49 (4.55)	29 (6.9)	4 (3.5)	16 (3.0)		
	Others		131 (12.17)	78 (18.4)	19 (16.5)	34 (6.3)		

Lifestyle	Washing hands after bowel movement	Yes	724 (62.28)	279 (66.0)	84 (73.0)	361 (67.1)	49.519	0.001⁣^∗^
		No	86 (7.99)	13 (3.1)	2 (1.7)	71 (13.2)		
		Often	266 (24.72)	131 (31.0)	29 (25.2)	106 (19.7)		
	Eating with hands	Yes	520 (48.32)	244 (57.7)	63 (54.8)	213 (39.6)	106.637	0.001⁣^∗^
		No	501 (46.56)	137 (32.4)	41 (35.7)	323 (60.0)		
		Often	55 (5.11)	42 (9.9)	11 (9.6)	2 (0.4)		
	Eating shellfish, salads, fruits, and vegetables	Yes	664 (61.71)	332 (78.7)	86 (74.4)	246 (45.7)	117.953	0.001⁣^∗^
		No	411 (38.19)	90 (21.3)	29 (25.2)	292 (54.3)		
	Eating ice cubes	Yes	669 (62.17)	285 (67.4)	76 (66.1)	308 (57.2)	11.164	0.004⁣^∗^
		No	408 (37.91)	138 (32.6)	39 (33.9)	230 (42.8)		
	Washing fruits before consumption	Yes	552 (51.49)	189 (44.7)	51 (44.3)	292 (54.3)	10.076	0.039⁣^∗^
		No	410 (38.10)	177 (41.8)	48 (41.7)	185 (34.4)		
		Often	134 (12.45)	57 (13.5)	16 (13.9)	61 (11.3)		
	Consumption of food and drinks offered by ambulant merchants	Yes	609 (56.59)	297 (70.2)	72 (62.6)	240 (44.6)	83.660	0.001⁣^∗^
		No	425 (39.49)	12 (29.3)	43 (37.4)	258 (48.0)		
		Often	42 (3.90)	2 (0.5)	0 (0)	40 (7.4)		

Symptoms	Digestive disorders	Yes	619 (57.52)	325 (77.0)	89 (77.4)	204 (37.9)	174.784	0.001⁣^∗^
		No	457 (42.47)	97 (23.0)	26 (22.6)	334 (62.1)		
	Stomach pain	Yes	426 (39.59)	235 (55.6)	71 (61.7)	120 (22.0)	135.881	0.001⁣^∗^
		No	650 (60.40)	188 (44.4)	44 (38.3)	418 (77.7)		
	Constipation	Yes	470 (43.68)	278 (65.7)	82 (71.3)	110 (20.4)	237.260	0.001⁣^∗^
		No	606 (56.31)	145 (34.3)	33 (28.7)	428 (79.6)		
	Diarrhea	Yes	458 (42.56)	279 (66.0)	70 (60.9)	109 (20.3)	219.925	0.001⁣^∗^
		No	618 (57.43)	144 (34.0)	45 (39.1)	429 (79.7)		
	Loss of appetite	Yes	424 (39.40)	252 (59.6)	75 (65.2)	97 (18.0)	207.105	0.001⁣^∗^
		No	652 (60.59)	171 (40.4)	40 (34.8)	441 (82.0)		
	Fever	Yes	580 (53.90)	328 (77.5)	95 (82.6)	157 (29.2)	264.796	0.001⁣^∗^
		No	496 (45.10)	95 (22.5)	20 (17.4)	380 (70.8)		
	Headache	Yes	505 (46.93)	292 (60.0)	79 (68.7)	134 (25.0)	208.941	0.001⁣^∗^
		No	571 (53.07)	131 (31.0)	36 (31.3)	403 (75.0)		
	Sore throat	Yes	259 (24.07)	13 (32.6)	34 (29.6)	87 (16.2)	37.202	0.001⁣^∗^
		No	817 (75.92)	285 (67.4)	81 (70.4)	451 (83.8)		
	Cough	Yes	356 (33.08)	227 (53.7)	66 (57.4)	103 (19.1)	144.790	0.001⁣^∗^
		No	680 (66.92)	196 (46.3)	49 (44.6)	435 (80.9)		
	Extreme exhaustion	Yes	325 (30.20)	239 (56.5)	62 (53.9)	24 (4.5)	338.546	0.001⁣^∗^
		No	751 (69.80)	184 (43.5)	53 (46.1)	514 (95.5)		
	Bleeding from the rectum	Yes	136 (12.63)	88 (20.8)	29 (25.2)	19 (3.5)	82.430	0.001⁣^∗^
		No	940 (87.37)	335 (79.2)	86 (74.8)	519 (96.5)		
	Pink spots	Yes	120 (11.15)	88 (20.8)	26 (22.6)	6 (1.1)	109.698	0.001⁣^∗^
		No	956 (88.85)	335 (79.2)	89 (77.4)	532 (98.9)		
	Diarrhea and constipation	Yes	183 (17.01)	135 (31.9)	37 (32.4)	11 (2.0)	170.676	0.001⁣^∗^
		No	893 (82.99)	188 (68.1)	78 (67.8)	527 (98.0)		

*Note:* “Informal sector,” a set of units producing goods and services with the main aim of creating jobs and income for the people concerned.

⁣^∗^Significant (*p* value < 0.05).

**Table 2:** Relationship between predictor variables and the two diseases following multivariate logistic regression analysis.

Characteristics of participants	Category	*S.* Typhi infection		*S.* Paraty*phi* infection	
		Odds ratio (95% CI)	*p* value	Odds ratio (95% CI)	*p* value
Sex	Male	0	0	0	0
	Female	1.428 (1.083–1.882)	0.012⁣^∗^	1.699 (1.103–2.618)	0.016⁣^∗^

Age range	1–10 years	10.845 (3.356–35.043)	0.001⁣^∗^	8.126 (0.955–69.155)	0.055
	11–20 years	4.259 (1.490–12.174)	0.007⁣^∗^	5.316 (0.705–40.078)	0.105
	21–30 years	4.030 (1.345–12.079)	0.013⁣^∗^	6.511 (0.940–45.095)	0.058
	31–40 years	1.270 (0.526–3.066)	0.594	5.594 (0.923–33.911)	0.061
	41–50 years	0.723 (0.294–1.777)	0.479	2.561 (0.449–14.594)	0.290
	≥ 51 years	0	0	0	0

Marital status	Married	0.289 (0.085–0.980)	0.046⁣^∗^	0.104 (0.016–0.677)	0.018⁣^∗^
	Single	0.295 (0.081–1.076)	0.064	0.133 (0.018–0.956)	0.045⁣^∗^
	Concubinage	1.103 (0.340–3.578)	0.870	0.250 (0.041–1.538)	0.135
	Divorced	1.629 (0.396–6.707)	0.499	0.287 (0.023–3.657)	0.337
	Widower	0	0	0	0

Profession	Students	0.502 (0.235–1.076)	0.076	1.885 (0.170–1.383)	0.135
	Pupils	0.442 (0.197–0.994)	0.048⁣^∗^	0.395 (0.125–1.246)	0.113
	Housewives	0.365 (0.151–0.885)	0.026⁣^∗^	0.232 (0.063–0.852)	0.028⁣^∗^
	Informal sector	0.527 (0.263–1.052)	0.069	0.420 (0.621–5.723)	0.070
	Civil servant	0	0	0	0

Level of education	No schooling	1.273 (0.604–2.682)	0.526	1.885 (0.621–5.723)	0.263
	Primary	1.271 (0.712–2.269)	0.418	1.109 (0.435–2.824)	0.828
	Secondary	1.285 (0.870–1.900)	0.208	0.984 (0.534–1.813)	0.959
	University	0	0	0	0

Source of water	Tap	0.412 (0.151–1.124)	0.083	0.254 (0.085–0.761)	0.014⁣^∗^
	Protected well	0.227 (0.079–0.648)	0.006⁣^∗^	0.148 (0.047–0.466)	0.001⁣^∗^
	Drilling (borehole)	0.765 (0.135–5.330)	0.762	0.551 (0.081–3.742)	0.542
	Others	0	0	0	0

Lifestyle	Washing hands after bowel movement	0.138 (0.072–0.700)	0.017⁣^∗^	0.167 (0.027–1.050)	0.056
	Eating with hands	0.085 (0.010–0.727)	0.024⁣^∗^	0.043 (0.005–0.391)	0.005⁣^∗^
	Eating shellfish, salads, fruits, and vegetables	3.319 (1.662–6.626)	0.001⁣^∗^	7.339 (3.105–17.348)	0.001⁣^∗^
	Eating ice cubes	2.044 (1.014–4.121)	0.046⁣^∗^	1.704 (0.769–3.776)	0.189
	Washing fruits before consumption	1.579 (0.526–4.743)	0.416	0.984 (0.299–3.241)	0.979
	Consumption of food and drinks offered by ambulant merchants	43.272 (8.866–211.190)	0.001⁣^∗^	21.805 (4.427–107.412)	0.001⁣^∗^

Symptoms	Digestive disorders	4.043 (1.972–8.292)	0.001⁣^∗^	3.305 (1.458–7.496)	0.004⁣^∗^
	Stomach pain	4.694 (2.282–9.657)	0.001⁣^∗^	4.274 (1.917–9.528)	0.001⁣^∗^
	Constipation	10.430 (4.986–22.241)	0.001⁣^∗^	7.613 (3.325–17.434)	0.001⁣^∗^
	Diarrhea	5.047 (2.499–10.194)	0.001⁣^∗^	5.992 (2.703–13.285)	0.001⁣^∗^
	Loss of appetite	5.800 (2.834–11.868)	0.001⁣^∗^	8.000 (3.588–17.838)	0.001⁣^∗^
	Fever	6.730 (3.267–13.863)	0.001⁣^∗^	6.807 (2.940–15.759)	0.001⁣^∗^
	Headache	6.265 (3.036–12.926)	0.001⁣^∗^	10.166 (4.435–23.303)	0.001⁣^∗^
	Sore throat	1.918 (0.840–4.372)	0.122	2.410 (0.975–4.960)	0.057
	Cough	3.297 (1.617–6.721)	0.001⁣^∗^	1.970 (0.892–3.348)	0.093
	Extreme tiredness	27.885 (10.720–72.536)	0.001⁣^∗^	24.249 (8.778–66.991)	0.001⁣^∗^
	Bleeding from the rectum	2.818 (1.040–7.635)	0.042⁣^∗^	2.306 (0.771–6.900)	0.135
	Pink spots	22.466 (4.471–112.888)	0.001⁣^∗^	41.527 (7.939–217.227)	0.001⁣^∗^
	Diarrhea and constipation	15.401 (5.355–44.288)	0.001⁣^∗^	8.250 (2.637–25.817)	0.001⁣^∗^

⁣^∗^Significant (*p* value < 0.05).

**Table 3:** Antibiotic resistance profile of *S.* Typhi and *S*. Paratyphi isolates at Dschang Annex Regional Hospital in Cameroon.

Antibiotics	S. Typhi positive, *n* = 155			S. Paratyphi positive, *n* = 52			Chi-square test (*χ*^2^)	*p* value
	*S n* (%)	*I n* (%)	*R n* (%)	*S n* (%)	*I n* (%)	*R n* (%)		
Quinolones								
Ciprofloxacin	130 (83.9)	24 (15.5)	1 (0.6)	46 (88.5)	6 (11.5)	0 (0.0)	0.850	0.654
Levofloxacin	147 (94.8)	7 (4.5)	1 (0.6)	50 (96.2)	2 (3.8)	0 (0.0)	0.383	0.826
Pefloxacin	155 (100.0)	0 (0.0)	0 (0.0)	52 (100.0)	0 (0.0)	0 (0.0)	/	/
Ofloxacin	154 (99.4)	1 (0.6)	0 (0.0)	52 (100.0)	0 (0.0)	0 (0.0)	0.337	0.562
Nalidixic acid	72 (46.5)	30 (19.4)	53 (34.2)	34 (65.4)	6 (11.5)	12 (23.1)	5.626	0.060
Penicillins								
Amoxicillin	49 (31.6)	8 (5.2)	98 (63.2)	17 (32.7)	5 (9.6)	30 (57.7)	1.437	0.487
Amoxicillin + clavulanic acid	59 (38.1)	53 (34.2)	43 (27.7)	17 (32.7)	19 (36.5)	16 (30.8)	0.493	0.782
Ampicillin	12 (7.7)	30 (19.4)	113 (72.9)	1 (1.9)	10 (19.2)	41 (78.8)	2.284	0.319
Aminoglycosides								
Streptomycin	88 (56.8)	5 (3.2)	62 (40.0)	25 (48.1)	4 (7.7)	23 (44.2)	2.496	0.287
Gentamycin	22 (14.2)	23 (14.8)	108 (69.7)	6 (11.5)	10 (19.2)	36 (69.7)	0.426	0.888
Amikacin	13 (8.4)	11 (7.1)	131 (84.5)	4 (7.7)	10 (19.2)	38 (73.1)	6.298	0.043
Cephalosporins								
Ceftriaxone	130 (83.9)	18 (11.6)	7 (4.8)	44 (84.6)	5 (9.6)	3 (5.8)	0.269	0.874
Cefotaxime	134 (86.5)	20 (12.9)	1 (0.6)	45 (86.5)	7 (13.5)	0 (0.0)	0.000	0.987
Tetracyclines								
Tetracycline	46 (29.7)	7 (4.5)	102 (65.8)	14 (26.9)	6 (11.5)	32 (61.5)	3.269	0.195
Doxycycline	51 (32.9)	13 (8.4)	91 (58.7)	20 (38.5)	5 (9.6)	27 (51.9)	0.733	0.693
Minocycline	46 (29.7)	0 (0.0)	109 (70.3)	16 (30.8)	2 (3.8)	34 (65.3)	0.465	0.505
Phenicols								
Chloramphenicol	54 (34.8)	16 (10.3)	85 (54.8)	15 (28.8)	4 (7.7)	33 (63.5)	1.206	0.547
Sulfonamides								
Trimethoprim	87 (56.1)	20 (12.9)	48 (31.0)	31 (59.6)	7 (13.5)	14 (26.9)	0.305	0.896
Sulfamethoxazole	19 (12.3)	7 (4.5)	129 (83.2)	6 (11.5)	3 (5.8)	43 (82.7)	0.145	0.930
Co-trimoxazole	92 (59.4)	7 (4.5)	56 (36.1)	23 (44.2)	3 (5.8)	26 (50.0)	3.621	0.177

Abbreviations: *I*, intermediary; *R*, resistance; *S*, sensitive.

**Table 4:** Resistance profile of *S*. Typhi and *S*. Paratyphi isolates among patients at Dschang Annex Regional Hospital in Cameroon.

Types of resistance	*Type of isolates*	Number of isolates	Resistance (%)
R3	*S.* Typhi	21	13.54
	*S.* Paratyphi	6	11.53

R4	*S.* Typhi	59	38.06
	*S.* Paratyphi	25	48.07

R5	*S.* Typhi	51	32.90
	*S.* Paratyphi	14	26.92

R6	*S.* Typhi	16	10.32
	*S.* Paratyphi	6	11.53

R7	*S.* Typhi	3	1.93
	*S.* Paratyphi	0	0.00

*Note:* R3–R7, number of antibiotic classes to which a given isolate was resistant.

## Data Availability

The datasets used to support the findings of this study are available from the corresponding author upon request.
